# Microbiological Analysis of Surgeons’ Hands in a Public Hospital in São Luis, Maranhão State, Brazil: A Cross-Sectional Study

**DOI:** 10.3390/microorganisms11081895

**Published:** 2023-07-27

**Authors:** Artur Serra Neto, Sirlei G. Marques, Maria Rosa Q. Bomfim, Silvio G. Monteiro, Rosangela C. de Souza, Rodolfo A. Nunes

**Affiliations:** 1 Departamento de Cirurgia Geral, Hospital Universitário da Universidade Federal do Maranhão (HUUFMA), São Luís 65020-070, Brazil; 2 Departamento de Planejamento de Gestão da Qualidade e Vigilância em Saúde, Hospital Universitário da Universidade Federal do Maranhão (HUUFMA), São Luís 65020-070, Brazil; sirlei.marques@huufma.br; 3 Departamento de Biologia Molecular, Universidade Ceuma (UNICEUMA), São Luís 65075-120, Brazil; mrqbomfim@gmail.com; 4 Departamento de Biologia, Universidade Federal do Maranhão (UFMA), São Luís 65080-805, Brazil; silvio.gm@ufma.br; 5 Departamento de Medicina, Universidade Federal do Maranhão (UFMA), São Luís 65080-805, Brazil; rosacipriano91@gmail.com; 6 Departamento de Cirurgia Geral, Faculdade de Ciências Médicas, Universidade do Estado do Rio de Janeiro (UERJ), Rio de Janeiro 20550-900, Brazil; rodolfoacatauassu@yahoo.com.br

**Keywords:** hands, antisepsis, surgeons, surgical sites, microbiology

## Abstract

Antisepsis of the hands of medical personnel is one of the most important steps in the process of patient care, since direct contact can cause the cross-transfer of potentially pathogenic microorganisms at surgical sites. This study aimed to analyze the prevalence of microorganisms on the hands of 131 surgeons in a university hospital before the surgical procedure. Swabs were collected from each clinician’s hands before and after handwashing. The samples were placed in a transport medium and immediately delivered to a private clinical analysis laboratory from São Luis-Maranhão. The microorganisms were identified by ionization source mass spectrometry and matrix-assisted laser desorption (MALDI-TOF), and antibiotic susceptibility tests (AST) were performed using the Vitek2 and Phoenix-BD automated system. The results showed a high frequency (100%) of microorganisms before handwashing, but after surgical antisepsis, the rate dropped significantly (*p* < 0.05) to 27.5%. The gram-positive species most detected were *Staphylococcus* spp. and *Micrococcus luteus*, representing 83.9%, followed by gram-negative species, *Stenotrophomonas maltophilia*, *Acinetobacter baumanii*, *Pseudomonas aeruginosa*, *Pseudomonas gessardi*, *Pantoea septica*, *Serratia marcescens*, and *Burkholderia lata.* The effectiveness of hand antisepsis was 72.5%, demonstrating that surgeons’ hands are an important source of microorganisms that can cause infections in hospitalized patients in different care settings.

## 1. Introduction

The significance of hand antisepsis for patient safety is not a new concept. Ignaz Philip Semmelweis, in 1847, established an important correlation between medical care and a higher maternal risk of puerperal fever, since the rates were much lower when parturients were assisted by midwives, who wash their hands frequently throughout the procedure [1]. Since then, several studies have correlated and demonstrated that the transmission and dissemination of microorganisms through the hands of health professionals has considerably impacted the occurrence of health care-associated infections (HAIs) (also referred to as “nosocomial” or “hospital” infections) [1,2,3,4,5,6,7,8,9,10]. Among the infections potentially transmitted by the hands of professional staff are surgical site infections (SSIs), which can occur during the surgical procedure, even in the operating room. These infections represent between 0.5% to 3% of adverse effects in surgical patients, increasing complications and hospitalization time [11,12].

Operating rooms and ICUs in general are considered critical areas for patient care due to the structural characteristics of the facilities and the presence of several equipment, including air conditioners, monitoring devices, secretion aspirators, catheters and probes, defibrillators, and pulmonary ventilators, among others. In addition, management by the health team in these areas can strongly influence care outcomes [13,14,15,16,17]. The operating room, for example, is an environment favorable to the spread of microorganisms that may be present on surfaces and/or equipment [14], and, depending on the intervention and the medical specialty, it may require multidisciplinary health professionals, including anesthesiologists, nurses, and surgeons. Thus, the greater the number of individuals in the operating room, the more likely biosafety norms could be violated or disregarded. Breaking patient safety protocols can lead to errors that were previously considered avoidable, including the incorrect administration of medications and team attitudes that contribute to surgical site infection [16,17]. On the other hand, patients of ICUs are at high risk of acquiring HAIs because they are usually immunologically compromised, and depending on their individual needs, they may be submitted to different invasive procedures [15].

HAIs are a serious public health problem that have increased considerably in recent years, and their occurrence is one of the most common adverse events in hospital care. It has negative impacts on the health of patients hospitalized in various sectors, especially those occupying intensive care unit (ICU) beds [2,3]. Furthermore, HAIs impose a significant financial burden on public funds, health insurance companies, patients, and their family members. It requires a prolonged hospitalization time, exposing the patient to potential risks and complications, which significantly increase morbidity and mortality rates [18,19]. HAIs affect millions of patients worldwide every year, with rates ranging from 7% to 10% [20,21,22,23]. These variations may be attributed to the implementation of patient safety behaviors and practices at different levels within each healthcare institution, as well as the socioeconomic conditions of each country. In affluent countries, for instance, where these institutions receive substantial financial support, the incidence rates vary between 3.5% and 12%. However, in developing countries where health is not prioritized, the rates range from 5.7% to 19.2% [19,24,25,26].

Some important factors may be associated with the high prevalence of HAIs in the hospital environment, including a long period of hospitalization; major surgical procedures; use of invasive instruments; indiscriminate use of antibiotics; conditions inherent to the patient, such as the existence of chronic diseases (diabetes, skin wounds); characteristics related to patient care sectors, such as intensive care units (ICUs); and use of inadequate hand hygiene techniques by visitors, nursing professionals, intensive care physicians, and surgeons [8,23]. In addition, patient infection can also occur through invasive procedures, such as intravenous drug administration, catheter and drain insertion, percutaneous tracheostomy, the use of prosthetic devices, endotracheal intubation, mechanical ventilation, oral routes, and surgery [1,8,23,25,27].

The set of factors described above demonstrates that the hospital environment offers specific conditions for circulation and prolonged persistence for many species of pathogenic microorganisms, including gram-negative (GN) bacteria with multidrug resistance (MDR) profiles to drugs used in clinical practice [27,28]. Studies have shown that the main species detected in HAIs are gram-positive microorganisms, especially members of the *Staphylococcus* genus, such as methicillin-resistant *Staphylococcus aureus* (MRSA), and other gram-negative species, including *Pseudomonas aeruginosa*, *Acinetobacter baumannii*, *Klebsiella pneumoniae*, and some members of the carbapenem-resistant/carbapenemase-producing *Enterobacteriaceae* family (e.g., *Escherichia coli*, *Enterobacter* spp., *Serratia* spp.) [27,28,29]. In addition to bacteria, several species of fungi have been isolated from samples of hospitalized patients; mainly those in ICUs, where among the most isolated are the yeast species (*Candida*), mainly *C. albicans*, *C. parapsisolis*, *C. glabrata*, and *Trichosporon asahii*, an emerging yeast. Other fungal species have also been detected, such as *Cryptococcus neoformans*, *Aspergillus fumigatus*, *Penicillium*, *Cladosporium*, and *Fusarium* [27,29,30,31,32,33]. All of these pathogens are involved in an increase in morbidity and mortality rates, length of hospital stay, and financial burden for patients, families, and health systems [24,27,34,35,36,37].

It is important to emphasize that outbreaks and even sporadic or continuous transmission of pathogenic microorganisms with MDR profiles can occur through the hands of health professionals to hospitalized patients, especially those in ICUs [33]. Thus, the contaminated hands of these professionals act as important vehicles for transporting pathogenic microorganisms to susceptible patients [2,3,8,21,22].

On the other hand, each person has a transient microbiota that changes over time, which is dependent on environmental conditions, availability of nutrition and/or stage of growth and health, or even the diurnal rhythms of the hosts [38,39]. Knowledge by the healthcare professional of the transient microbial flora on their hands and the hospital environment can help them to select the appropriate detergent, thereby reducing the prevalence of pathogenic microorganisms both in the community and in healthcare settings [38,40]. In addition to this transient microbiota, health workers can be contaminated during the handling of patients colonized by pathogens or by microorganisms that can remain viable on equipment, on the surfaces of bedroom and bathroom furniture, on instrument carts, bed rails, bed surfaces, bedside tables, intravenous pumps, and handwashing faucets [27,28,41,42,43]. 

Handwashing is a prerequisite for reducing the occurrence of HAI [38]. Therefore, several authors have reinforced its importance and drawn attention to the significant drop, around 70%, in the incidence and prevalence rates of HAIs [1,22,23,25,38]. Although no protocol guarantees the elimination of 100% of the microorganisms present on the hands of health professionals, the simple act of washing hands by visitors, nurses, and other members of health care teams may reduce the risk of transmission of HAIs [1,44]. During this process, transient flora that colonize the surface of the skin can be removed, and the resident microbiota of the skin of the hands and forearms of the professionals who participate in the surgeries can be reduced [45].

The antisepsis performed by the ordinary citizen and/or visitor to the hospitalized patient is simpler than that used by health professionals, since the latter must do so judiciously, before and after direct or indirect contact with patients, following the protocol adopted by the hospital [46]. In Brazil, surgeons have used the guidelines for safe surgery of the World Health Organization [40]. Despite the wide use of these guidelines, the complete elimination of microorganisms from the hands of health professionals remains a major challenge, implying the need for changes in habits and educational measures [38].

In this context, the main objectives of this study were to evaluate the efficiency of the handwashing procedure by surgeons working in a public hospital in the city of São Luis, Maranhao, Brazil; as well as to identify the main factors that affect the efficiency of antisepsis on the hands of surgeons and determine the antimicrobial susceptibility profile of the species that most persist on the hands of surgeons after surgical handwashing. Additionally, this study aimed to analyze the level of knowledge and application of the six international patient safety standards by each evaluated surgeon.

## 2. Materials and Methods

### 2.1. Type of Study and Place of Sample Collection

This was an observational analytical epidemiological study of the cross-sectional type with an applied nature and a quantitative and qualitative approach that was carried out between May and September 2022, in the University Hospital of the Federal University of Maranhao (HUUFMA). This hospital is a teaching and research center that trains health professionals for undergraduate students in nursing, pharmacy, medicine, nutrition, dentistry, psychology, occupational therapy, physiotherapy, speech therapy, social work, and related areas. It is also a state reference hospital for highly complex procedures in the areas of cardiovascular surgery, traumato-orthopedics, neurosurgery, video-laparoscopy, nephrology, transplants, high-risk pregnancy, bariatric surgery, lithotripsy, hemodynamics, audiometry, magnetic resonance imaging, hepatology, ophthalmology, and surgical oncology.

#### Eligibility Criteria, Inclusion Criteria, Non-Inclusion Criteria, and Limitations of the Study

The HUUFMA has a population of 230 health professionals, including surgeons and resident physicians who work in surgical centers. Considering a 95% confidence level and a 5% margin of error, the number of professionals that would be included in the study was calculated to be 144. We increased the number to 150 to try to divide them equally between the different specialties of the hospital. The selection was based on convenience, considering the number of surgeons per specialty; for example, the hospital has more general surgeons, orthopedists, and urologists, so they were more heavily represented. Of this total, 19 were excluded for presenting incomplete data, leaving 131 surgeons eligible for the survey.

The sample inclusion criteria consisted of being a surgeon working at HUUFMA surgical centers and agreeing to participate in the research. Additionally, they were required to have performed at least one surgical procedure during the investigated period and to have practiced surgical hand antisepsis.

The non-inclusion criteria were surgeons who, despite having completed the questionnaire, chose to withdraw from the research without any prompting. Additionally, samples that were not correctly identified or were damaged during transport were also excluded.

The quantitative variables collected were age, the doctor’s time since graduation, the number of colonies before and after antisepsis, and the number of species before and after antisepsis in relation to the two groups, such as sex (male or female) or use of antisepsis products (chlorhexidine or others), in relation to the three levels of education (specialization, master’s, and doctorate).

As a research limitation, we acknowledge that our study only focused on a public hospital and did not include sampling from various other types of hospitals. Therefore, the sample population is small, and some specialties have a limited number of specialists, such as otorhinolaryngologists.

### 2.2. Sample Collection with Sterile Swabs

Before commencing sample collection, each surgeon was informed about the research objectives and was required to sign an informed consent form. They were also asked to complete a structured sociodemographic questionnaire, which included questions about their age, sex, place of residence, and the number of hospitals where they work.

Sample collection was carried out from May to December 2022 on the days scheduled for surgeries, always before the first surgery of the day and in accordance with the institution’s surgical schedule. The lavatories (1 and 2) existing inside the surgical center were used exclusively for the scrubbing of the surgeons’ hands. To avoid bias in the research, collections were always made by the same researcher. In addition, no instructions were given to the surgeons to evaluate their usual behaviors and precisely ascertain their efficiency from this procedure.

Samples from both hands of the surgeons chosen to participate in the research were collected before (A) and after (D) brushing, using sterile cotton swabs soaked in sterile 0.9% saline solution, in the following sequence: finger pulp, interdigital region, palms, back of hands, and wrist. Immediately after collection, the swabs were placed in Stuart’s transport medium and identified as sample A (before brushing) and sample D (after brushing), each surgeon with identified by a number.

Afterward, the sequence, duration, and product used for brushing were also observed. The duly identified samples were stored in thermal boxes in accordance with the criteria set by the National Health Surveillance Agency (ANVISA) of Brazil. They were then transported to the microbiology sector of a private laboratory in São Luís, Maranhão, Brazil for the purpose of isolating, identifying, and testing the susceptibility of the microorganisms. Every week, a water sample from each washroom was also sent for microbiological analysis and seeded in a culture medium (R2A) from Probac do Brasil^®^, which is specifically designed for counting heterotrophic microorganisms in water samples.

### 2.3. Microbiological Identification and Antimicrobial Susceptibility Tests

After the samples arrived at the microbiology laboratory, the swabs were tracked in the internal system and sent for sowing on blood agar and Sabouraud dextrose agar (for the isolation of bacteria and fungi, respectively). The initial inoculum for the isolation of microorganisms present in the swab/Swart medium was prepared in Eppendorf tubes with 900 µL of sterile 0.5% saline solution, with 100 µL of initial inoculum added. Serial decimal dilutions were made up to 10^6^ dilutions. Then, 100 µL of the last three dilutions were removed and seeded in three 10 µL sterile plastic handle plates. This procedure was performed for all samples collected, before (A) and after (D) the hand antisepsis process. Subsequently, the blood agar plates were incubated in a bacteriological oven at 35.5 ± 1 °C for 24–48 h, and the Sabouraud tubes were incubated at 28 ± 1 °C and 37 ± 1 °C for up to 15 days.

To conduct tests for the identification of bacterial and fungal samples, recent cultures of bacteria (18 to 24 h) and yeast fungi (72 h) were used. The identification of isolated microorganisms was performed using mass spectrometry with the Microflex-Bruker Daltonics/BioTyper™ (MALDI-TOF) equipment (Bruker Daltonics GmbH and Co. KG—Bremen, Germany). The equipment was calibrated initially with the Bruker Bacterial Test Standard (BTS) as recommended by the manufacturer. A portion of the microorganism colony to be identified was then extracted using a sterile toothpick and transferred to the appropriate spot on the Maldi plate. A thin smear was made within one of the spots on the plate, and this process was carried out in duplicate. After drying in the open air, 1 µL of the Matrix solution HCCA (α-cyano-4-hydroxycinnamic acid) (Sigma-Aldrich, Burlington, MA, USA)^®^ was added. The organic solvent present in the matrix solution facilitated the extraction of proteins, particularly ribosomal proteins found in the sample. After the crystallization of the matrix, the sample preparation was completed, and the plate was inserted into the equipment for reading. The analysis was performed by MALDI mass spectrometry (Matrix-Assisted Laser Desorption/Ionization Time-Of-Flight).

The laser in the MALDI-TOF spectrometer irradiated the matrix–sample compound, and the matrix was rapidly evaporated, releasing proteins with a positive charge (soft ionization technique). These ions were electrostatically accelerated over a short distance and entered the flight tube with a velocity that depended on the mass of each microorganism to be identified. As different proteins have varying masses, their respective ions arrived at the detector at different times (time of flight). By measuring the time (in the nanosecond range) between the pulsed acceleration and the corresponding detector signals, it was possible to accurately determine the velocity and convert it into an exact molecular mass. Data were obtained using specific software on the equipment, and the mass spectra of the samples were compared with the mass spectra of a bank of known microorganisms using MALDI-TOF. The manufacturer’s identification criteria stated that a score of ≥2.0 was considered reliable for species-level identification.

To determine the susceptibility profile of the isolated bacteria in relation to antimicrobials, the Phoenix-BD equipment (Becton and Dickinson, Franklin Lakes, NJ, USA) and the AST-NMIC-406 extended panels were used for Gram-negative bacilli, while the AST-PMIC-89 panel was used for Gram-positive cocci. To determine the susceptibility profile of the yeasts, the automated system Vitek2-CC4 (bioMérieux, Inc., Durham, NC, USA) with the AST-YS07 cards was used. For the vancomycin and polymyxin susceptibility test, the broth microdilution technique was employed to determine the minimum inhibitory concentration. For multi-resistant microorganisms, the test was repeated using the Muller-Hinton and E-Test^®^ bioMerieux gradient strips to confirm the minimum inhibitory concentration (MIC) or resistance. The results were interpreted based on the readings obtained from the cutoff points and analyzed according to the parameters established by the Brazilian Committee on Antimicrobial Susceptibility Testing (BrCast)/European Committee on Antimicrobial Susceptibility Testing guidelines. The results were reported using the categories Sensitive, Intermediate, and Resistant. Quality control was performed weekly using reference strains from the American Type Culture Collection, as recommended by the BrCAST/EUCAST Internal Control document [47].

### 2.4. Statistical Analysis

Data analysis was performed using IBM SPSS Statistics 22 software [48]. Initially, descriptive statistics of the analyzed variables were performed using frequency tables and graphs. For numerical variables, the minimum, maximum, mean, and standard deviation estimates were calculated. Subsequently, the normality of the quantitative variables (age, formation time, number of colonies before and after asepsis, number of species before and after antisepsis) was evaluated using the Shapiro–Wilk test, as all of them had a *p*-value < 0.05, indicating that they did not have a normal distribution and, therefore, were evaluated using non-parametric tests. Comparison of two independent groups, such as the effect of the surgeon’s sex (male or female), the effect of the use of antiseptic (chlorhexidine or others), and the effect of brushing time (2 min or more than 2 min) was performed using the Mann–Whitney test. When comparing three or more groups, for example, the effect of the three levels of education (specialization, master’s, and doctorate) performed by the non-parametric test by Kruskal–Wallis.

The evaluation of the presence or absence of microorganisms before and after hand antisepsis was performed using the McNemar nonparametric test. Before–after comparison for paired data on the number of microorganism species was performed with the Wilcoxon signed rank test. The association of nominal variables (presence or absence of microorganisms after antisepsis) in relation to the two groups, such as sex (male or female) or use of antisepsis products (chlorhexidine or others), or in relation to the three levels of education (specialization, master’s, and doctorate), the Chi-square test of independence was performed. The selection of the main risk factors related to the prevalence of cases of contamination after washing the hands of surgeons was initially carried out by univariate logistic analysis (not adjusted) considering as a selection criterion all variables that presented a value of *p* < 0.20. Subsequently, multivariate (adjusted) logistic regression was performed with these selected variables to estimate the Odds Ratios (OR), with 95% confidence intervals. In all tests, the significance level (α) used was 5%; that is, differences were considered significant when *p* was <0.05.

### 2.5. Ethical Aspects of Research

This project was submitted and approved by the Research Ethics Committee of the University Hospital of the Federal University of Maranhão under opinion number 2.638.389/2018. All participants read, approved, and signed the informed consent form.

## 3. Results

### 3.1. Sample Characterization

In this study, 131 surgeons were evaluated for microbiological analysis of their hands before and after the antisepsis procedure. The sample consisted of 108 (82.4%) men and 23 (17.5%) women, chosen randomly from the surgical clinical staff of HUUFMA. Initially, to each physician was explained how the research would be conducted. After obtaining signed free and informed consent forms for ethical considerations, a structured self-administered questionnaire with closed questions was answered to obtain sociodemographic data and qualitative variables for subsequent statistical analysis, as outlined in the materials and methods section.

### 3.2. Microbiological Identification of Microorganism Species

The results of the microbiological analyses of the samples collected before handwashing showed that there was growth of microorganisms in all 131 samples, with a frequency of 283 isolates belonging to 67 different species. Of this total, 217 (76.7%) isolates were gram-positive bacteria belonging to 36 different species; 55 (19.4%) isolates were gram-negative bacteria belonging to 24 species, and 11 (3.9%) isolates were fungi distributed in seven different species (Table 1).

A predominance of gram-positive microorganisms was observed on the evaluated hands; the presence of 133 isolates distributed across 11 different species of the *Staphylococci* genus corresponded to 61.3% of the isolates, followed by 44 strains of *Micrococcus luteus*, which represented 20.26% of the isolates recovered before washing hands (Table 1).

Microbiological analyses of the swabs obtained from the hands of the 131 surgeons after the antisepsis process showed that no microorganism grew from 88 (72.5%) of the samples, but there was growth of 49 colonies in samples from the hands of 36 (27.5%) surgeons (Table 2). The identification performed by MALDI-TOF of these colonies detected 26 different species of microorganisms after the handwashing procedure. Observed were 25 strains of *Staphylococcus* spp., representing 73.5% of all microorganisms isolated. Of these, 24 isolates were coagulase-negative (CoNS); the most found were *S. warneri*, *S. capitis*, and *S. cohnii*. Furthermore, *Micrococcus luteus* was also prevalent, representing 12.2% of the total. Other gram-positives found were *Bacillus cereus*, *Kocuria kristinae*, *Dermacoccus nishinomyaensis*, *Brevibacterium ravenspurgense*, and *Bacillus simplex* (Table 2).

Among the gram-negative species, two isolates of *Stenotrophomonas maltophilia* were recovered after antisepsis. We found the presence of mixed infections in 8 (30.8%) of these samples, where it was possible to verify the predominance of gram-positive microorganisms on the hands of these surgeons (Table 2). In addition, some fungal species were identified before and after the antisepsis process, including *Candida parapsilosis*, *Aspegillus versicolor*, and *Trichosporon asahii* (Table 1 and Table 2). 

### 3.3. Antimicrobial Susceptibility Tests

The results of antimicrobial susceptibility tests for gram-positive microorganisms isolated from the hands of physicians after surgical antisepsis obtained by the BD Phoenix™ automated system (AST-Pmic89) showed sensitivity for all isolates evaluated, and only *S. aureus* presented an intermediate profile of sensitivity to levofloxacin. The results of the automated system Vitek2CC4 (AST-N408) for the susceptibility profile for glucose-fermenting and nonfermenting gram-negative microorganisms showed that *Pseudomonas aeruginosa* was resistant to the carbapenems imipenem and meropenem; *Pseudomonas gessardi* was resistant to aztreonam; and *Serratia marcescens* was resistant to ampicillin (Table 3).

It is important to note that no growth of microorganisms was observed in any of the 12 water samples collected from the two lavatories that were used for handwashing before surgical procedures. 

### 3.4. Statistical Analysis

Statistical analysis of the frequency distribution of the sociodemographic variables of the 131 surgeons analyzed showed that 108 (82.4%) were men and 23 (17.5%) were women. The McNemar test for the presence and absence of microorganisms on the surgeons’ hands before and after surgical antisepsis showed a very high frequency (100%) before washing, and after antisepsis, this frequency dropped significantly (*p* < 0.05) to 27.5%. The non-parametric Mann–Whitney test was conducted to analyze the numerical variables (number of species of microorganisms and colonies before and after antisepsis) in relation to the surgeon’s sex. The results indicated that there was no significant difference (*p* > 0.05), suggesting that the surgeon’s sex does not have an influence on the asepsis of the hands of surgeons.

Concerning age, time since graduation, specialties, and titration, the results are shown in Table 4. The age groups with the highest number of surgeons were from 30 to 39 years (32.8%) and 40–49 years (22.9%). Most of them, 49 (37.4%), were between 1 and 5 years after graduation; only 14 (10.7%) were more than 30 years post-graduation. As for the specialty, the two most prevalent were general surgery (42, 32.1%) and orthopedics (27, 20.6%). Regarding their degrees, 73 (55.7%) were specialists, and only 7 (5.3%) had a doctorate (Table 4).

**Figure 1:**
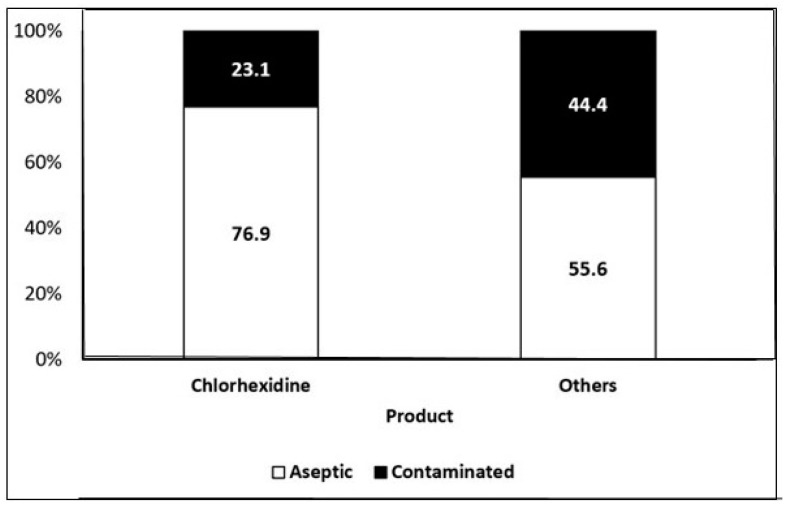
Association of frequency of contamination in relation to the asepsis product. (chlorhexidine and other products).

Data from clinical variables on how to perform the surgical antisepsis process (brushing time, sequence, products used), knowledge of international safety goals, and risk of infection in surgery, safe surgery protocol, and occurrence of surgical site infection (ISC) are presented in Table 5. The results obtained showed that the most, 100 (76.3%), of the surgeons spent 3 to 5 min in the brushing process, 73 (55.7%) performed the technique correctly, 104 (79.4%) used chlorhexidine, and 91 (69.5%) used the safe surgery protocol. The results of analyses of contamination in relation to the antiseptic product (chlorhexidine and other products) was conducted using the Chi-square test of independence. It was found that the use of chlorhexidine significantly reduced contamination (Chi-2 = 4.91, *p* < 0.05) to about 48% the frequency of contamination when compared to other products (Figure 1). 

**Figure 2:**
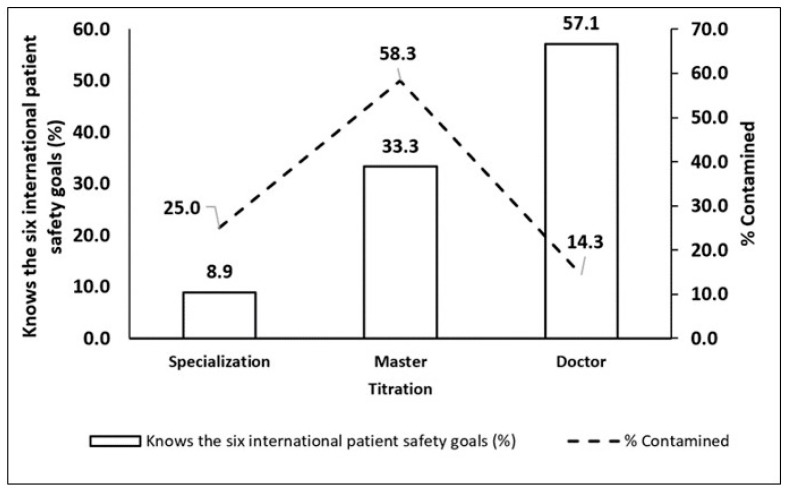
Association of frequency of contamination and knowledge of the six international goals for patient safety in relation to titration.

The Chi-square test was used to assess whether the surgeon with the highest level of qualification had a greater understanding of international patient safety standards and whether they were more meticulous in the surgical asepsis procedure. The results showed a significant association (Chi-2 = 17.2, *p* < 0.05) of dependence and the surgeon’s degree when evaluating knowledge of the six international goals for patient safety. The higher the degree, the greater the knowledge of the six goals. A significant association was also observed between the presence and absence of contaminants after asepsis (Chi-2 = 6.69, *p* = 0.035) in relation to the surgeons’ titles. Surgeons with doctoral qualifications had fewer contaminants after hand asepsis (14.3% of contaminants) compared to with a master’s degree (58.3%). However, those who had only specialization had 25% less contaminants (Figure 2).

Of the 100 surgeries performed by the 131 physicians after antisepsis of the hands, laparoscopic cholecystectomy was the most common surgical procedure, accounting for 15 (11.4%) (Table 6).

Table 7 shows that significant differences (*p* < 0.05) were found in measurements of the number of colonies and species of microorganisms after hand antisepsis in relation to titration, brushing time, and the use of antiseptic products.

**Figure 3:**
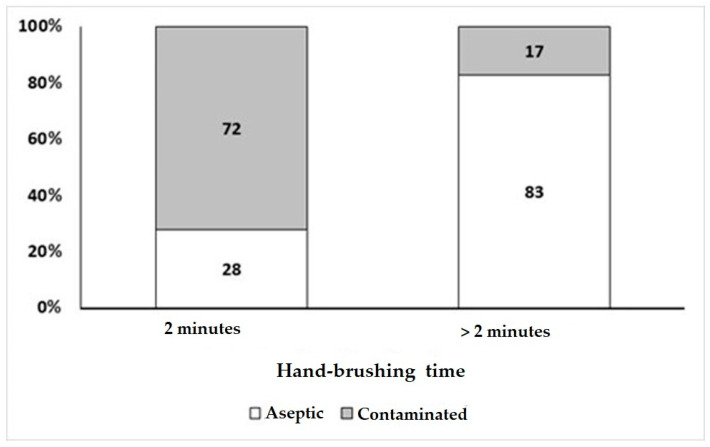
Relationship between contamination frequency and hand-brushing time.

The Chi-square test for independence showed a significant association (*p* < 0.05) between titration, use of the antiseptic product, and time spent brushing hands, with the frequency of hand contamination after antisepsis (Table 8). The surgeons with the lowest frequency of contamination were those who were doctors (14.3%), who used chlorhexidine (23.1%), and who brushed their hands for more than 2 min (83%) (Figure 3).

Table 9 shows the analysis of risk factors for contamination after hand antisepsis performed using logistic regression. In the univariate analysis (unadjusted), the independent variables titer, use of antiseptic products, and brushing time in hand hygiene were selected (*p* < 0.20) for the multivariate analysis (adjusted). In the multivariate analysis, it was verified that brushing time equal to 2 min increases the risk of contamination by 12 times when compared with more than 2 min (OR = 12.15 CI95% = 4.3–34.1 *p* = 0.001).

**Figure 4:**
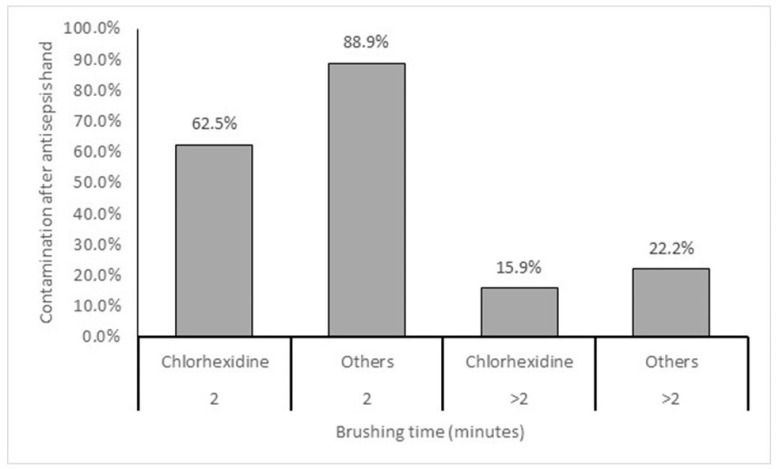
Frequency of contamination after hand antisepsis in relation to the type of antiseptic product and brushing time.

The use of 2% chlorhexidine significantly reduces (*p* < 0.05) hand contamination after antisepsis, but brushing time longer than 2 min had a more expressive effect in reducing hand contamination of surgeons after antisepsis. antisepsis. (Figure 4).

## 4. Discussion

In this study, we evaluated the microbiological profile of the hands of resident physicians, specialists, masters, and doctors before and after surgical antisepsis. Despite the existence of a mandatory protocol for daily use in the researched hospital, the frequency rates of microorganisms on the hands of the evaluated surgeons were very high at both timepoints: before handwashing, (100%) and after this procedure (27.5% of the doctors still had microorganisms on their hands). These results are consistent with several studies that have indicated the potential transmission of microorganisms, both pathogenic and non-pathogenic, at any given time within the hospital environment. This transmission often occurs through the hands of healthcare professionals, especially when proper hand hygiene is not followed during routine interventions [5,9,21,46,49,50]. These studies have demonstrated the effectiveness and highlighted the importance of hand hygiene for the overall health of the population, especially for individuals who utilize health services. The World Health Organization, for example, has made significant contributions to the dissemination and implementation of strategies aimed at promoting a culture of hand hygiene, particularly among health professionals [36,40,51].

Handwashing has contributed significantly to the drop in the transmission of infectious agents. Child death rates from respiratory and diarrheal diseases have declined, with a decrease of 21 percent and 30 percent, respectively, for children under five. It has also contributed to a decrease in sepsis and HAIs [51]. A longitudinal study conducted in Finland from May 2013 to December 2020 evaluated the effects of direct hand hygiene among nurses and doctors in medical and surgical wards. The study observed that when doctors, surgeons, and nurses followed the handwashing monitoring program, the incidence rate of HAI decreased from 15.9 to 13.5 per 1000 patient-days (*p* < 0.0001) [52]. In a punctual prevalence survey carried out by Magill et al. [53], comparing the prevalence of HAI between 2011 and 2015, during a period of national attention to prevention in the United States, the authors observed that the risk of patients having HAI was 16% lower in 2015 compared to 2011.

We found a high prevalence of gram-positive bacteria before (61.3%) and after (20.26%) the antisepsis procedure on the evaluated professionals’ hands; especially species of the *Staphylococci* genus can theoretically be explained, as these are part of the microbiota of the human skin and mucosa [54]. However, their permanence in the hands of surgeons after antisepsis is a worrying fact because these microorganisms are important opportunistic pathogens that have shown high rates of involvement in infections in hospitalized patients [37,38]. This finding is consistent with the results of microbiological research conducted on pathogens that come from patients in the intensive care unit of a hospital in the Iraqi city of Erbil. They found a higher prevalence of gram-positive bacteria (83.1%), with *Staphylococcus aureus* accounting for 78.6% of isolates. *Streptococci* (33.3%) and *enterococci* (28.6%) were also found in significant numbers. Lower rates were found for gram-negative bacteria (16.9%), including *Escherichia coli* (19%), *Pseudomonas aeruginosa*, and *Proteus* spp. (4.8%). The surgical center had the highest contamination rate of 35.6%, while the ICU had a rate of 21.4% [55].

We isolated several gram-positive bacteria of clinical importance after the aseptic process of the surgeons’ hands, with emphasis on some species of the genus *Staphylococcus*; among them *Staphylococcus warneri*, *Staphylococcus capitis*, *Staphylococcus hominis*, *Staphylococcus hemolyticus*, and *Micrococcus luteus*. Other species also isolated include *Bacillus cereus*, *Bacillus simplex*, *Brevibacterium ravenspurgense*, *Dermacoccus nishinomyaensis*, and *Kocuria kristinae* (Table 2). These findings are like those obtained by Liu et al. [49], who found the presence of different species of *Kocuria* spp., *Brevibacterium* spp., *Dermacoccus* spp., *Bacillus simplex*, and *B. cereus* before and after hand antisepsis in anesthesiologists and in the operating room. This sporulated *bacillus* can be present in soil and food and can easily be taken to the hospital environment through the skin of health workers; it has been implicated in nosocomial infections [56]. On the other hand, *Bacillus simplex* is an environmental microorganism whose habitat is the soil; a recent report documented the occurrence of infection in a traumatic injury [57].

The presence of different species of microorganisms, such as *Staphylococcus warneri*, *Staphylococcus capitis*, *Staphylococcus hominis*, *Staphylococcus hemolyticus*, *Micrococcus luteus*, and *Stenotrophomonas maltophilia*, on the hands of surgeons after antisepsis calls attention to one more complicating factor for the hospital unit in question, and it is worth noting that eight professionals from the same team of doctors work at another public hospital in São Luis-MA. A similar study was carried out by Avestan et al. [9] to investigate contamination on the hands of nurses working in different types of wards in a hospital in Iran. Before the hand antisepsis process, they found that 22 (40.76%) of the isolates were coagulase-negative staphylococci, and 6 (11.12%) were *S. aureus*. After hand washing, the number of these species was reduced, 1 for *S. aureus* and 3 for CoNS species. A Brazilian study carried out in a neonatal unit on the impact of 70% alcohol gel on the bacteria present on the hands of health workers, before and after the use of this product, showed that there was bacterial growth in 104 (48.6%) of the samples collected before of hand hygiene, with a reduction in the load to 52 (24.3%) microorganisms after antisepsis with alcohol gel. Coagulase-negative species were more isolated before and after the antisepsis process [58]. Szemraj et al. [59] emphasize the importance of coagulase-negative staphylococci, including *S. hemolyticus*, *S. hominis*, *S. warneri*, and *S. simulans*, as etiological agents of serious infections.

Other interesting findings of the present study include the isolation of *Brevibacterium ravenspurgense*, a microorganism that may be present on human skin. No reports of this species being involved in HAIs were found. The detection of this species in the present study may serve as a warning for infection control committees. We found only one report of a previous case involving the commensal *B. casei* in a child with acute leukemia [60]. Furthermore, the bacteria *Kocuria kristinae*, which can colonize the skin, mucous membranes, and oropharynx, causing invasive infections in immunocompromised patients, particularly in children, has been isolated [58,59,60,61,62]. Some studies have highlighted the clinical importance of some species of the genus *Kocuria* [61,62]; for example, *Kocuria* spp. has been isolated from endophthalmic infections [63]. *K. kristinae* has been involved in infections in different anatomical sites, including the urinary tract [64]; pneumonia [65]; catheter-associated bacteremia [66]; endocarditis [67,68,69]; and osteoarthritis [70], among other cases reported in a review study [71].

We also recovered some species of gram-negative bacteria from surgeons’ hands after the antisepsis process, including *Stenotrophomonas maltophilia*, *Acinetobacter baumanii*, *Pantoea septica*, *Pseudomonas aeruginosa*, *Pseudomonas gessardi*, *Serratia marcescens*, and *Burkholderia lata*. Additionally, three species of fungi were identified: *Aspergillus versicolor*, *Candida parapsilosis*, and *Trichosporon asahii*. All of these results are important, including the isolation of the gram-negative bacillus *S. maltophilia*, that has been considered an emerging opportunistic pathogen of worldwide concern that has been associated with high rates of morbidity and mortality among immunocompromised patients with malignant tumors, diabetes, and the use of immunosuppressants drugs [72], and its occurrence has been underestimated; it is multiresistant to different classes of antibiotics, making the treatment of infections challenging [72,73,74].

Interestingly, here we found that the gram-negative nonfermenting *bacilli P. aeruginosa* and *P. gessardi* showed resistance profiles to carbapenems, specifically imipenem and meropenem, and to aztreonam, respectively. However, the glucose-fermenting bacilli *Serratia marcescens* was resistant to ampicillin. Among the species of the genus *Pseudomonas*, *P. aeruginosa* is considered an important cause of HAIs and is one of the major resistant gram-negative pathogens [37]. Large outbreaks of infections caused by *Acinetobacter baumannii* and *Pseudomonas aeruginosa* have been implicated in HAIs worldwide. These bacteria are considered opportunistic pathogens that carry genes conferring multiresistance profiles to several classes of antimicrobials [75,76]. In addition, *Candida* species, which have emerged as opportunistic pathogens, including *C. parapsilosis*, as well as other fungi such as *Aspergillus versicolor*, *Rhodothorula mucilaginosa*, and *Trichosporon asahii*, are involved in invasive infections in immunocompromised patients. This is particularly true for patients with cancer, those undergoing organ transplants, or those receiving immunosuppressant drugs after transplantation [77]. 

Regarding the findings above, an epidemiological survey conducted by Suetens et al. [29] investigated the prevalence of HAI and antimicrobial resistance in intensive care hospitals and long-term care institutions across 28 countries in Europe, involving 1735 hospitals. They found a prevalence of 5.9% of HAI in the total sample, with a variation of 4.4% in primary care hospitals and 7.1% in tertiary hospitals. The highest prevalence, 19.2%, was observed among patients hospitalized in intensive care units, where at least one occurrence of HAI was reported. The most found microorganisms were *E. coli*, *S. aureus*, *Klebsiella* spp., *Enterococcus* spp., *P. aeruginosa*, *C. difficile*, coagulase-negative *staphylococci*, *Candida* spp., *Enterobacter* spp., and *Proteus* spp., respectively. An overall profile of 6.2% resistance to carbapenems was found among species of the *Enterobacteriaceae* family.

Considering the results described in Table 2, it is emphasized that they cannot be ignored. Interestingly, a significant portion of the species isolated here are not commonly found in the hospital environment. Some reports describe them as opportunistic microorganisms. Another highly relevant aspect is the fact that human hands are considered carriers of a substantial number of microorganisms from numerous species. In this scenario, when verifying that surgeons themselves are bringing potential pathogens with opportunistic nature into the hospital environment, it raises concerns about an increase in the occurrence of HAI by these microorganisms. In this scenario, it is important to note that the presence of the microorganisms on the hands of surgeons implies the need for greater care during antisepsis, since this procedure does not eliminate all microorganisms from the microbiota on the surgeon’s hands or on the adjacent areas of the surgical site on the patient [78].

Regarding the importance of hand washing in reducing the incidence of HAIs, a study conducted during the COVID-19 pandemic showed that strict hand hygiene reduced the rate of HAIs in a tertiary hospital in Pakistan. They suggested that this was because the number of professionals participating in the handwashing program increased significantly [79]. Although the pandemic caused great fear among health care professionals of infecting their family members, it was expected that adherence to the handwashing program by these professionals would increase significantly, but this was not observed in practice [80]. One study showed that hand hygiene adherence during the pandemic period was low among health professionals, even in hospitals that had adequate infrastructure and antiseptics. The authors postulated that this low adherence rate may be related to the respondents’ low knowledge of the actual importance of this individual and collective protective measure [81].

Concerning the different times used by the surgeons to perform hand antisepsis, we found that the majority, 76.3%, spent 3 to 5 min, six of them spent more than 5 min, while that 19.1% performed the wash in 2 min (Table 5). It is important to note that the researcher responsible for collecting the samples did not interfere or give any instructions about the procedure. Thus, data obtained during surgical antisepsis accurately reflect the amount of time usually spent by each research participant. Our intention was to evaluate the surgeons’ typical behaviors to accurately determine their effectiveness in preventing contamination. Regarding physicians who spent 2 min on asepsis, the multivariate statistical analysis showed that brushing time equal to 2 min increases the risk of contamination by 12 times when compared with more than 2 min. Furthermore, we observed that the use of 2% chlorhexidine reduced hand contamination after antisepsis, but brushing time longer than 2 min had a more expressive effect in reducing hand contamination of surgeons after antisepsis (Figure 4). Although there are some contradictions regarding the use of artifacts, such as a brush or sponge, in hand antisepsis, as well as the antiseptic product [82,83,84], most of them follow the traditional technique that uses 2% chlorhexidine [82], as it has longer-lasting effects [84]. As for brushing time, the average brushing time is 2 min [85], although ANVISA recommends 3 to 5 min. This work agrees with ANVISA because when the brushing time is longer than 2 min, it significantly reduces the colony-forming unit (CFU) [83,85,86,87]. On the other hand, one of the disadvantages of using a brush is the probability of causing injuries to the surgeon’s skin, increasing the risk of contamination or discomfort, which is why it is suggested not to use this artifact or to the reduction in brushing time or just rubbing the hands with the antisepsis product [82].

Handwashing is a simple procedure, but it requires attention and adherence to the sequence recommended by the World Health Organization, which should be adopted by all health professionals. As far as the washing time is concerned, it may depend on the exfoliation agent formula; generally, 3 min can be as effective as 5 min exfoliation [40]. It is noteworthy that, naturally, surgeons use sterile gloves, but if some are not able to perform the correct antisepsis of the hands when putting on the gloves, they could contaminate the gloves during this process and, in this case, become a source of infection for their patient during the surgical procedure. In addition, after two hours of surgery, approximately 35% of the gloves have small perforations, allowing the entry of body fluids [40]. 

In this study, we observed through paired analysis that all antiseptic products used for washing reduced the microbial flora of the hands; but the surgeons who used chlorhexidine had a lower frequency of surgical contamination when compared to those who used water plus povidone or povidone iodine. The chlorhexidine was the most efficient in hand antisepsis than other products, such as povidone-iodine (Figure 1). 

Some studies have shown that there are no differences between povidone and chlorhexidine in terms of effectiveness in reducing the bacterial flora of the skin; however, povidone-iodine has iodine-related clinical risks; it is more expensive, and the period of action is reduced in the presence of organic compounds [88]. On the other hand, chlorhexidine does not lose its effectiveness when exposed to blood and has a longer disinfecting effect on the skin, so it is often used for hand washing, and has shown lower rates of surgical infection [88,89,90].

Additionally, the statistical analysis of sociodemographic data showed a low number of physicians with a doctorate; most of them were between 30 and 39 years old, and they had a short time since graduation, from 1 to 5 years. Surgeons with doctoral qualifications stood out statistically regarding the understanding of the six International Patient Safety (Figure 2). Furthermore, this group showed the lowest frequency of contamination those who were doctors (14.3%), who used chlorhexidine (23.1%), and who brushed their hands for more than 2 min (83%) (Figure 3 and Table 8). Regarding this result, no data were found in the literature that correlated the degree of the surgeon with his effectiveness in hand washing, or with the highest level of knowledge of the six biosafety standards. On the other hand, the results of the analysis to verify the occurrence of the influence of the surgeons’ sex on the effectiveness of antisepsis showed that it did not appear to have a significant influence on the efficiency of hand antisepsis; this result is different those obtained by Liu et al. [49], where female anesthesiologists performed better hand antisepsis than male anesthesiologists and observed fewer colony-forming units and fewer species on their hands. However, our result was also different from what was found in another study, in which women tended to clean their hands more than men [38].

In the context described above, it is highlighted the hospital environment involves highly complex dynamics that make it challenging to determine the exact source of infection outbreaks [3,18,19,42]. However, the occurrence of HAIs is a preventable event in up to 70% of cases [22]. Despite this complexity it is important to point out that patient safety begins in the admission process and extends throughout the provision of health care. According to the World Health Organization (WHO), patient safety is defined as the “prevention of errors and adverse events associated with patient care” [40].

## 5. Conclusions

Antisepsis of the hands of health professionals working in a surgical center is one of the most important steps in the health care process, as failure to follow this procedure can cause the transfer of potentially pathogenic microorganisms to surgical sites, which is one of the most important goals of the patient safety program. In the present study, microbiological evaluation of surgeons’ hands before and after surgical washing showed that most members of the medical team did not perform antisepsis efficiently. Consequently, isolation revealed a high incidence of various microorganisms, most of which are considered opportunistic in healthcare-associated infections. Prominent among these were gram-positive cocci and bacilli, gram-negative bacilli, and yeasts. Among the most commonly found cocci are several species of the genus *Staphylococcus* spp. followed by *Micrococcus luteus*; in addition, other emerging and clinically relevant gram-positive species were isolated in the hospital environment, including *Kocuria kristinae*, *Dermacoccus nishinomyaensis*, *Brevibacterium ravenspurgense*, *Bacillus simplex*, and *Bacillus cereus*. With regard to gram-negative species isolated after surgical washing of surgeons’ hands, in addition to the species *A. baumanii*, *Pantoea septica. P. aeruginosa*, *P. gessardi*, and *S. marcescens*; pathogens frequently cited in the literature, *Stenotrophomonas maltophilia* and *Burkholderia lata* were also found to be emerging as new microorganisms in the hospital environment. The presence of these opportunistic microorganisms with pathogenic potential on the hands of these surgeons may contribute to the occurrence of adverse events such as surgical site infections and other healthcare-associated infections, especially when barrier mechanisms, such as the use of sterile gloves, are breached. We also observed that 2% chlorhexidine was more efficient than water plus povidone and povidone iodine; that the longer the hand washing time, the greater the chances of reducing the amount and number of species of microorganisms on the hands; and that surgeons with a doctorate degree have greater knowledge of the six international standards safety and also carry out hand washing more efficiently. Thus, the time spent washing hands, the type of antiseptic product used, and the correct execution of antisepsis are important factors that directly interfere with the final result of the surgical washing process. These findings are worrying because the hospital studied is a teaching hospital that serves as a regional reference for the training of physicians, specialists, and nurses. In addition, due to its character as a training facility for health professionals, there is a high turnover of physicians in the surgical environment. The findings of the present study showed that several urgent needs must be met at the investigated hospital, including the implementation of policies and measures for training and monitoring of knowledge and applicability of the six international patient safety standards; the use of a set of measures for continuous training of medical and nursing teams, with emphasis on strengthening the handwashing procedure; the monitoring of adverse event indicators; and the implementation of a permanent education program focused on patient health and the fight against nosocomial infections. 

## Figures and Tables

**Table 1:** Frequency distribution of microorganism species isolated before surgical antisepsis of the hands.

Gram-Positive	n.	%	Gram-Negative	n.	%	Fungus	n.	%
*Aerococcus viridans*	1	0.45	*Acinetobacter baumanii*	5	9.11	*Aspegillus versicolor*	2	18.20
*Bacillus simplex*	1	0.45	*Acinetobacter ursingii*	2	3.63	*Candida haemulonii*	1	9.10
*Brevibacterium casei*	2	0.90	*Acinetobacter variabilis*	2	3.63	*Candida parapsilosis*	4	36.30
*Brevibacterium celere*	7	3.21	*Aspergillus fumigatus*	1	1.82	*Pichia kudriavzevii (C. krusei)*	1	9.10
*B. ravenspurgense*	1	0.45	*Citrobacter sedlakii*	1	1.82	*Rhodothorula mucilaginosa*	1	9.10
*Corynebacterium casei*	1	0.45	*Enterobacter cloacae*	2	3.63	*Trichosporon asahii*	1	9.10
*C. minutissimum*	1	0.45	*Enterococcus faecalis*	1	1.82	*Trichosporum japonicum*	1	9.10
*C. simulans*	1	0.45	*Klebsiella pneumoniae*	1	1.82			
*Dermacoccus nishinomiyaensis*	2	0.90	*Methylobacterium radiotolerans*	1	1.82			
*Kocuria kristinae*	1	0.45	*Moraxela osloensis*	2	3.63			
*Kocuria marina*	2	0.90	*Moraxella spp*	1	1.82			
*Micrococcus luteus*	44	20.26	*Pantoea dispersa*	2	3.63			
*Oceanobacillus onocorhynchi*	1	0.45	*Pantoea septica*	2	3.63			
*Penicillium spp*	1	0.45	*Pseudomonas aeruginosa*	1	1.82			
*Rhodococcus equi*	1	0.45	*Pseudomonas extremorientalis*	1	1.82			
*Bacillus cereus*	7	3.21	*Pseudomonas gessardi*	1	1.82			
*Bacillus clausii*	1	0.45	*Pseudomonas stutzeri*	2	3.63			
*Bacillus megaterium*	1	0.45	*Serratia marcescens*	2	3.63			
*Bacillus pumilus*	1	0.45	*Burkholderia lata*	1	1.82			
*B. amyloliquefaciens*	1	0.45	*Mixta calida (Pantoea callida)*	2	3.63			
*B. thermoamylovorans*	1	0.45	*Neisseria subflava*	1	1.82			
*Lactobacillus paracasei*	1	0.45	*Rhizobium radiobacter*	1	1.82			
*Staphylococcus aureus*	6	2.76	*Roseomonas mucosa*	5	9.11			
*S. arlettae*	1	0.45	*Stenotrophomonas maltophilia*	15	27.27			
*S. capitis*	12	5.52						
*S.caprae*	1	0.45						
*S. cohnii*	10	4.60						
*S. epidemidis*	40	18.40						
*S. haemolyticus*	19	8.75						
*S.hominis*	10	4.60						
*S. saprophyticus*	10	4.60						
*S. sciuri*	1	0.45						
*S. warneri*	23	10.59						
*S. xylosus*	2	0.90						
*Streptococcus parasanguinis*	1	0.45						
*Streptomyces nogalater*	2	0.90						
Total	217	100.0		55	100.0		11	100.0

Legend: n. = number of isolated.

**Table 2:** Distribution of the frequency of 49 species isolated from the hands of 36 surgeons who presented contamination after the surgical antisepsis process.

Species	Type	N.	%	Mixed Contamination	N.	%
*Acinetobacter baumanii*	gram-neg.	1	2.0	*S. warneri*, *M. luteus*, *K. kirstinae*	1	2.0
*Pantoea septica*	gram-neg.	1	2.0	*S. warneri*, *A. baumanii*, *M. luteus*	1	2.0
*Pseudomonas aeruginosa*	gram-neg.	1	2.0	*T. asahii. B. lata*, *M. luteus*	1	2.0
*Pseudomonas gessardi*	gram-neg.	1	2.0	*S. cohnii*, *M. luteus*	1	2.0
*Serratia marcescens*	gram-neg.	1	2.0	*S. xylosus*, *S. conhnii*	1	2.0
Burkholderia lata *	gram-neg.	1	2.0	*A. versicolor*, *C. parapsolosis*	1	2.0
*Stenotrophomonas maltophilia*	gram-neg.	2	4.0	*S. capitis*, *M. luteus*	1	4.0
	subtotal	8	16.3	*S. warneri*, *S. saprophyticus*	1	2.0
				subtotal	8	16.3
*Bacillus cereus*	gram-pos.	2	4.0			
*Bacillus simplex*	gram-pos.	1	2.0			
*Brevibacterium ravenspurgense*	gram-pos.	1	2.0			
*Dermacoccus nishinomyaensis*	gram-pos.	1	2.0			
Kocuria kristinae *	gram-pos.	1	2.0			
Micrococcus luteus *	gram-pos.	6	12.2			
Staphylococcus warneri *	gram-pos.	7	14.2			
*Staphylococcus aureus*	gram-pos.	1	2.0			
Staphylococcus capitis *	gram-pos.	6	12.2			
*Staphylococcus caprae*	gram-pos.	1	2.0			
Staphylococcus cohnii *	gram-pos.	3	8.2			
*Staphylococcus epidermidis*	gram-pos.	2	4.0			
*Staphylococcus haemolyticus*	gram-pos.	1	2.0			
*Staphylococcus hominis*	gram-pos.	2	4.0			
Staphylococcus saprophyticus *	gram-pos.	1	2.0			
Staphylococcus xylosus *	gram-pos.	1	2.0			
	subtotal	37	75.5			
Aspegillus versicolor *	fungus	1	2.0			
Candida parapsilosis *	fungus	2	4.0			
Trichosporon asahii *	fungus	1	2.0			
	subtotal	4	8.2			
	Total	49	100		8	100

Legend: Neg. = negative. Pos. = positive. * Mixed contamination.

**Table 3:** Antibiotic resistance profiles obtained by the automated Vitek2 system for gram-negative non-fermenting bacilli and glucose-fermenting bacilli isolated from surgeons’ hands after an antisepsis process.

Non-Glucose Fermenting Bacilli						Glucose-Fermenting Bacilli	
Microorganisms	*S. maltophilia* n = 2	*A. baumannii* n = 1	*P. aeruginosa* n = 1	*P. gessardi* n = 1	*Burkholderia lata* n = 1	*Pantoea septica* n = 1	*Serratia marcescens* n = 1
Antibiotics							
amikacin	-	Sus.	Sus.	Sus.	Na	Sus.	Sus.
ampicillin	-	-	-	-	-	-	Res.
aztreonam	-	-	-	Res.	Na	-	-
cefepime	-	-	Int.	-	Na	Sus.	Sus.
ceftazidime	-	-	Int.	Int.	Na	-	Sus.
ceftazidime-avibactam	-	-	-	Sus.	Na	-	-
ceftriaxone	-	-	-	-	Na	Sus.	Sus.
ciprofloxacin	-	Sus.	Int.	Int.	Na	Sus.	Sus.
gentamicin	-	Sus.	-	-	Na	Sus.	Sus.
imipenem	-	Sus.	Res.	Int.	Na	Sus.	Sus.
meropenem	-	Sus.	Res.	Sus.	Na	Sus.	-
piperacillin-tazobactam	-	-	Int.	Int.	Na	-	Sus.
Levofloxacin	Sus.	Int.	Int.	Int.	Na	Sus.	-
Sulfamethoxazole + trimethoprim	Int.	Int.	-	-	Na	Sus.	-

Legend: Na = Not applicable; Sus. = susceptible; Int. = Intermediary; Res. = Resistant.

**Table 4:** Frequency distribution of socio-demographic variables among the 131 interviewed surgeons.

Variables		Number	Percentage (%)
Age group	<30	27	20.6
	30–39	43	32.8
	40–49	30	22.9
	50–59	20	15.3
	60–69	8	6.1
	70 ou +	3	2.3
Time since graduation (years)	1–5	49	37.4
	6–10	25	19.1
	11–15	13	9.9
	16–20	9	6.9
	21–25	6	4.6
	26–30	15	11.4
	>30	14	10.7
Specialties	General surgery	42	32.1
	Orthopedics	27	20.6
	Urology	11	8.4
	Vascular surgery	9	6.9
	Digestive system	8	6.1
	Cardiac surgery	7	5.3
	Coloproctology	6	4.6
	Neurosurgery	6	4.6
	Head neck	4	3.0
	Maxillary mouth	3	2.3
	Plastic surgery	3	2.3
	Otorhinolaryngology	3	2.3
	Thoracic Specialties surgery	2	1.5
Titration	Resident	39	29.8
	Specialist	73	55.7
	Master	12	9.2
	Doctor	7	5.3

**Table 5:** Frequency distribution of clinical variables in surgical hand antisepsis by surgeons and safety goals.

Variables		n	%
Brushing time (minutes)	2	25	19.1
	3 to 5	100	76.3
	>5	6	4.6
Sequence	Correct	73	55.7
	Incorrect	58	44.3
Product	Water plus povidone	3	2.3
	Chlorhexidine	104	79.4
	Povidone iodine	24	18.3
Risk of infection in surgery	Contaminated	8	6.1
	Infected	7	5.3
	Clean	91	69.5
	Potentially contaminated	25	19.1
Knows the six international patient safety goals	In part	83	63.3
	No	30	23.0
	Yes	18	13.7
What is the goal *	Ignore	45	34.1
	1-Correctly identify the patient	70	53.0
	2-Improve communication between health professionals	6	4.5
	3-Improve safety in the prescription, use and administration of medications	9	6.8
	4-Ensure surgery in the correct intervention site, procedure, and patient	88	66.7
	5-Sanitize your hands to avoid infections	26	19.7
	6-Reduce the risk of falls and pressure ulcers	11	8.3
Responds to safe surgery protocol	No	31	23.7
	Yes	100	76.3
Surgical site infection (SSI)	Ignore	15	11.4
	No	91	69.5
	Yes	25	19.1

Legend. * = multiple answers.

**Table 6:** Frequency distribution of types of procedures performed after antisepsis of hands.

Type of Surgery	n	%	Type of Surgery	n	%
laparoscopic cholecystectomy	15	11.4	thyroidectomy	2	1.5
tibial osteosynthesis	7	5.3	wrist fracture	2	1.5
inguinal hernioplasty	6	5.3	valve replacement	2	1.5
appendectomy	6	3.8	abdominoplasty	1	0.8
prostatectomy	5		hand amputation	1	0.8
discectomy	4	3.0	brain aneurysm	1	0.8
carotid endarterectomy	4	3.0	maxillary antrostomy	1	0.8
laparotomy	4	3.0	spine arthrodesis	1	0.8
myocardial revascularization	4	3.0	hip arthroplasty	1	0.8
thigh amputation	3	2.3	knee arthroscopy	1	0.8
gastroplasty	3	2.3	thyroglossal cyst	1	0.8
hemorrhoidectomy	3	2.3	colectomy	1	0.8
femur osteosynthesis	3	2.3	pancreaticoduodenectomy	1	0.8
intestinal transit reconstruction	3	2.3	chest tumor excision	1	0.8
bone tumor resection	3	2.3	fasciotomy	1	0.8
tracheostomy	3	2.3	arteriovenous fistula	1	0.8
choledochotomy	2	1.5	cerebrospinal fluid fistula	1	0.8
hand debridement	2	1.5	mandibular fracture	1	0.8
leg debridement	2	1.5	hepatectomy	1	0.8
excision of saliva calculus	2	1.5	incisional hernia	1	0.8
brain tumor excision	2	1.5	lymphadenectomy	1	0.8
open fracture leg	2	1.5	mammaplasty	1	0.8
gastrectomy	2	1.5	mastectomy	1	0.8
hysterectomy	2	1.5	nephrectomy	1	0.8
shoulder arthroscopy	2	1.5	radius osteosynthesis	1	0.8
laparoscopic nephrectomy	2	1.5	transurethral resection of the prostate	1	0.8
wrist osteosynthesis	2	1.5	thoracoscopy	1	0.8
sinusotomy	2	1.5	urethroplasty	1	0.8

**Table 7:** Analysis of the number of colonies and the number of species after antisepsis of hands.

Variables		Nº of Colonies after Antisepsis				
		N	Means	SD	Test	*p*
Sex	Feminine	23	5.5	15.0	−0.42	0.677
	Masculine	109	5.4	18.7		
Titration	Specialization	112	4.5	16.4	6.67 ^a^	0.010
	Master	12	17.3	31.4		
	Doctor	7	0.7	1.9		
Brushing time (minutes)	2	25	17.6	31.1	−5.67	0.000
	>2	106	2.6	11.9		
knowledge of the six international goals for patient safety	No	113	5.4	18.5	−0.37	0.715
	Yes	18	5.6	16.3		
Aseptic product	Chlorhexidine	104	2.4	10.3	−2.77	0.006
	Others	27	17.2	32.3		
Variables		Nº of species after antisepsis				
		N	Means	SD	Test	*p*
Sex	Feminine	23	0.3	0.6	−0.61	0.541
	Masculine	109	0.4	0.8		
Titration	Specialization	112	0.4	0.8	6.72 ^a^	0.010
	Master	12	1.0	1.1		
	Doctor	7	0.1	0.4		
Brushing time (minutes)	2	25	1.2	1.2	−5.56	0.000
	>2	106	0.2	0.6		
knowledge of the six international goals for patient safety	No	113	0.4	0.8	−0.36	0.720
	Yes	18	0.4	1.0		
Aseptic product	Chlorhexidine	104	0.3	0.7	−2.26	0.024
	Others	27	0.7	1.2		

Legend. ^a^ Kruskal Wallis test.

**Table 8:** Association analysis between risk factors and contamination of surgeons’ hands after antisepsis of hands.

Variables		Contamination of Surgeons’ Hands after Antisepsis						
		No		Yes				
		n	%	n	%	Total	Chi-2	*p*
Sex	Feminine	18	78.3	5	21.7	23	0.43	0.512
	Masculine	78	71.6	31	28.4	109		
Titration	Specialization	84	75.0	28	25.0	112	6.69	0.035
	Master	5	41.7	7	58.3	12		
	Doctor	6	85.7	1	14.3	7		
Aseptic product	Chlorhexidine	80	76.9	24	23.1	104	4.91	0.027
	Others	15	55.6	12	44.4	27		
Knowledge of the six international goals for patient safety	No	81	71.7	32	28.3	113	0.29	0.591
	Yes	14	77.8	4	22.2	18		
Hand-brushing time (minutes)	2	7	28.0	18	72.0	25	30.73	0.001
	>2	88	83.0	18	17.0	106		

**Table 9:** Logistic regression analysis (univariate and multivariate) of the rate of contamination of surgeons’ hands after antisepsis of hands.

Variable Independent	Univariate			Multivariate		
	*p*	OR	CI 95%	*p*	OR	CI 95%
Sex (Feminine)	0.514	0.70	0.24–2.05			
Titration (Specialization)	0.129	0.46	0.17–1.25	0.502	0.56	0.10–3.06
Hand-brushing time (2 min)	0.001	12.57	4.58–34.50	0.001	12.15	4.31–34.19
Knows the six safety measures (No)	0.592	1.38	0.42–4.52			
Antisepsis product (Chlorhexidine)	0.030	0.38	0.15–0.91	0.170	0.49	0.17–1.36

## Data Availability

All data analyzed during this study are included in this published article. The raw data of this study are available from the author A.S.N. upon reasonable request.
